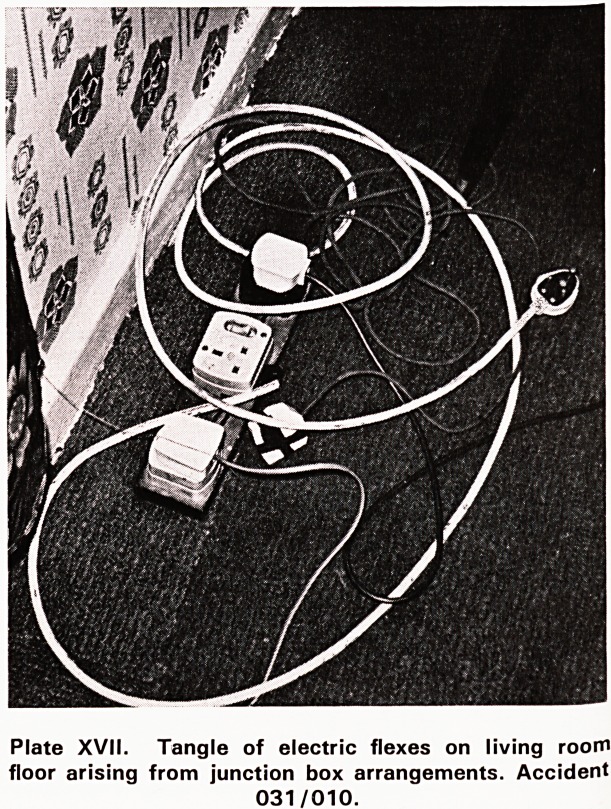# Investigating Accidents in the Home
*A paper read to The Bristol Medico-Chirurgical Society January 1972.


**Published:** 1972-10

**Authors:** C. P. de Fonseka, J. L. Roberts

**Affiliations:** Clinical Assistant Royal United Hospital, Bath and Research Fellow Medical Research Division Health Education Council; Health Economist, Medical Research Division Health Education Council and Lecturer in Health Economics, Dept. Public Health, University of Bristol


### Investigating Accidents in the Home*

**C. P. de Fonseka, M.S., F.R.C.S.**
Clinical Assistant Royal United Hospital, Bath and Research Fellow Medical Research Division Health Education Council
and
**J. L. Roberts, B.Soc.Sc., M.Sc., A.H.A.**
Health Economist, Medical Research Division Health Education Council and Lecturer in Health Economics, Dept. Public Health, University of Bristol.

### METHODS OF INVESTIGATION ON SITE

The value of on-the-spot study of accidents as a research method has been well established for road traffic, for aircraft and for railway accidents (States and States 1968, Robertson, Mclean and Ryan 1966, Jamieson et al. 1971, Kolbuszewski et al. 1969, Hadon, Suchmann and Klein 1964, De Haven 1942). The methods were developed in these fields as a result of the need for closer scientific scrutiny of growing accident problems at a time when the other major causes of premature death and disability were declining because of advances in public health and medical care. As accidents were studied more closely underlying causal factors were detected in the physical and psychological environment; remedies of re-design of equipment and structures and of re-training and re-education of key people—drivers, pilots, navigators—were begun. The basis for developing the prevention programmes was on-the-spot investigation.

The most satisfactory method of studying an event is to observe and measure its characteristics as it occurs. This cannot be done with accidents because of their comparative rarity and their unpredictability in time and space. But if we can set up a fast information system so that when an accident occurs we can as quickly as possible visit the scene with a team of trained investigators, observe the results of the accident, examine the phenomena involved, interview witnesses, attempt to determine the sequence of events leading up to the accident and to the injury and damage, we may be able to construct a sufficiently realistic model of the factors involved. This model may then be used in developing general theories of causation and for prevention in other similar situations.

Further laboratory experiment and control surveys may be necessary to test the model and subsequently community experiments or field trials must be done to explore the cost and value of the preventive measures. By giving an event the label 'Accident' we generally consider it an event of momentary duration which happens suddenly "out of the blue". Chance or the gods determine its action and its victim probably considers it "just one of those things". Primitive man, who witnessed lightning, attempted to explain it in terms of the super-natural; but with knowledge of electricity we can now better appreciate the events leading up to a flash, which is only a single incident in a successive chain of physical events, involving cloud, rain and electricity extending both into the pre-flash and post-flash periods of time; and we can explore mathematically the costs and benefits of lightning conductors on buildings of known height and value. Our model must embrace measures of predictability, preventability and cost and benefit. A similar approach to other so-called accidents—for example in transport—has resulted in changes in our understanding of human environmental control (De Haven 1952) and in particular has established the important conceptual distinction between accident causation and injury causation. The prediction of fatal injury in aircraft accidents has depended on detailed comparative study of survivors and non-survivors in given crashes and careful identification of injury causation (Hasbrook 1953).

A collision between two cars appears to the casual bystander as a sudden momentary event. However, the pre-collision factors could extend for a long period into the past, quite unknown to the witness. The factors may include poor vehicle maintenance, alcohol taken by the driver, a family quarrel or a poor road surface. All these factors require investigation and study before scientific prevention can be envisaged. Information is obtained by studying the scene and the vehicle, and interviewing those involved. The sooner these measures are carried out the more reliable and complete the data that will be collected.

High speed photographic studies of staged crashes have demonstrated that during collision there is a rapid sequence of events, each of which may leave a clue. The main purpose of visiting the scene is to gather these clues. For example, if a forehead hits a windscreen and shatters it, particles of glass may be embedded in the wound and bloodstained fragments of glass may be left on top of the facia. These facts when gathered will be proof that the windscreen was the cause of that particular injury. The following two examples are derived from the Road Accident Study at Birmingham University in which one of the present writers (CPdeF) was involved (Kolbuszewski et al. 1969). They show how a model of injury causation can be established, including the sequence in which the individual events took place.

Plate V shows the imprint of the lips, in lipstick, of a woman driver of the Mini Saloon shown in Plate

*A paper read to The Bristol Medico-Chirurgical Society January 1972.*

**Plate V. Lipstick imprint of driver's mouth on bonnet of Mini immediately in front of the windscreen frame. Note fragments of glass still held in rubber surround.**

VI which crashed into a lamp post. The imprint is on the top of the bonnet in front of the windscreen and steering wheel, which proves that her face hit that area. The presence of the steering wheel and the windscreen makes that part of the bonnet normally inaccessible to the driver's face. The force of the impact however pushed the steering column to the off-side. This would have allowed the driver's face to strike and shatter the screen. We can therefore conclude that the steering column must have moved aside *before* the driver went forward, thus establishing the sequence of the two separate events.

Plates VII and VIII illustrate the results of an impact between a motor cyclist's knee and the rear light cluster of a stationary Ford Corsair. The motor cyclist was admitted to hospital with a simple fracture of the lower part of the leg. In the region of the knee he had no injury at all, not even a contusion of the skin. Plate IX shows the dented car and the perfect fit of the knee and the leg against the distorted metal as it must have been at the time of impact. The inward distortion of the metal amounted to about 240 mm at the maximum point. The knee did not get injured in this instance because all the energy of the impact was absorbed by the deforming metal. There was therefore no energy left to cause injury to the knee. Injury is but the visible result of energy dissipation within tissues: the basis of safety in re-design of car interiors should be to absorb energy and thereby prevent injury to occupants in a crash. We do not suggest that rear light clusters of Ford Corsairs were specifically designed to prevent injury to the knees of motor

**Plate VI. Exterior of Mini which hit a lamp post showing damaged near-side front.**

**Plate VII. Off-side rear-light cluster of stationary Ford Corsair photographed on site, showing damage caused by impact of motor cyclist's knee.**

**Plate VIII. X-ray of left leg of motor-cyclist showing fracture of the lower part of the tibia and fibula.**

**Plate IX. Simulation of the position of the motor cyclist's knee at the moment of impact.**

cyclists; but the details of this incident, which were collected during on-the-spot investigation, give a very clear illustration of the energy dissipation model. The fracture sustained by the lower part of the leg (Plate VIII) can also be explained on the same basis. The illustrations show that the lower leg must have come into contact with the sloping bumper below the deformed metal. The metal bumper has not been deformed (Plate VII); it has in fact not absorbed any energy. Therefore the energy of the impact between the leg and the bumper was dissipated in the leg, causing the simple fracture of the tibia and fibula. The overall effect of the pattern of energy absorption by the deforming metal in this particular impact was the conversion of what would otherwise have been a very severe compound fracture of the knee into an easily treated simple fracture of the leg.

Immediately after a road accident all the participating objects and people are at the scene. But with the lapse of time and as the scene is cleared evidence gets scattered and destroyed. The opportunity to reconstruct events rapidly recedes. The advantages of on site investigations before the scattering of evidence are so great with road accidents that it was decided to explore a similar technique with accidents in the home.

### THE APPLICATION OF THE ON-THE-SPOT METHOD TO HOME ACCIDENTS

The decision to use the on-the-spot method of investigation for home accidents in Bristol was made with the full awareness that considerable modification to detail would be required. Whereas road accidents occurred in public places which could be visited at any time without permission, homes were quite a different matter. We had to compromise between the research need to get to the scene as quickly as possible and the social need to respect the privacy of the home. Accidents may be associated in people's minds with some apportionment of blame which can raise delicate issues for the investigator. In other cases the victim may have classified the event as carelessness —for which subsequent investigation is a waste of time and public money. A cautious approach with initial assurances by the investigator that he is not concerned to assess blame and that all accidents however trivial or inexplicable are important to the research contributes greatly to the extent of co-operation received from the public.

The permission necessary to enter the home has to be obtained from the householder or another responsible member of the family. Many accidents occur when the householder is away at work and involve his wife or children. In the case of elderly persons sustaining injury, after they had been removed to hospital there was often nobody in the house. There was, in addition, the delay of at least a few days involved in the system of notification, which it was not always possible to overcome. There were many such factors which contributed to making it impracticable to visit the accident site immediately after the accident. From experience with road accidents we expected the rapid obliteration of clues, such as happened with broken glass and skid marks, by clearing up procedures. It was therefore felt that the delay in visiting home accidents would also lead to the loss of much relevant material. However after only a little fieldwork it became apparent that in home accidents clues and objects do not disappear so quickly. Broken crockery, damaged saucepans and such objects are discarded to dustbins where they remain for some days and we succeed in retrieving such items on many occasions before the local dustman calls. Sometimes we fail, but when big articles are involved, such as cookers and washing machines, even if damaged, they are neither discarded nor repaired for quite some time. Therefore it is possible in many instances to look at the damage and even to test the apparatus in its damaged condition on our visit some days after the accident. Hazards in the structure of the house such as polished steps and irregular floors, furniture and fittings, frayed mats and carpets remain and few householders appeared sufficiently motivated even after an accident to change them. Thus the unavoidable delay in visiting home accidents was somewhat compensated by the relative stability of the scene compared with road accidents.

### THE COLLECTION OF DATA AT THE SCENE

With road accidents there were three main areas which required studying, the road environment needing highway engineering skills, the vehicles needing mechanical engineering skills and the human element involving medical, psychological and social science skills. Many years of research in these areas had established the variables that were probably relevant and methods of measurement were readily available. Further there were many basic similarities between individual road accidents, especially with regard to the vehicle. All vehicle interiors had features in common, all drivers sat behind steering wheels and all front passengers faced windscreens and facias. Road surfaces were essentially similar with individual variation. This similarity helped data collection. In contrast to this the environment in which accidents occur in homes is extremely varied. Wooden staircases and concrete steps may have many features in common but they differ from each other in many respects not previously adequately recorded in the accident literature. Both these environments differ completely from living rooms and bedrooms. The garden, the cellar, the attic and the bathroom provide four widely differing environments in which accidents occur and for which there is little available literature to provide a starting point for a scientific study. Data collection is further complicated by the variety of objects which are involved; knives and tin openers, crockery and glassware, laundry boilers and kettles, cookers and irons, shoes and slippers are but a few. In each case we have had to learn a fresh nomenclature before the specialist literature and manufacturing system which produced the product could be understood. In addition there are the power distribution systems involving electricity and gas and all the necessary wires and pipes. Data relating to all these different technologies has to be collected.

When we started collecting data we did not know for certain how frequently these various special technical problems would be involved. The Medical Research Division did not have the specialist personnel with knowledge in all these areas. As the research progressed and accidents in these various areas were encountered, specialists were contacted and advice obtained. For this purpose it was essential that the data recorded at the scene should be available in the greatest detail to the specialist and classified in a form recognising the critical points of differentiation he would need to know. As we can only partially succeed in this without all the specialists on site, much of the observation of the accidents must be essentially pre-scientific, identifying cases where specialists may be relevant to undertake more detailed investigations.

### OBJECTIVES

Our first objective was to establish a measure of frequency, severity and cost of those home accidents which resulted in a person making use of medical care services. We also wished to classify these events in a way which would identify the most frequently occurring types of accidents with a view to predicting high risk groups and establishing the likely applicability of the many existing prevention hypotheses. This would give us a baseline for further study. Previous studies of home accidents have included little on site investigation and our study was designed to begin to fill the gap that Backett's review of the field in 1965 had described (Backett 1965) and to provide the information of frequency, severity and cost that the Medical Commission on Accident Prevention had called for in their 1970-1971 Report.

### THE STUDY AREA

We chose an area of nine contiguous electoral wards in North East Bristol, which extended from the central city area, with its characteristics of decaying 19th century property and commercial office building programmes, out to the limit of the city boundary, at the edge of the green belt of Gloucestershire and traversing the varied area of industrial suburbs, enveloped villages, ribbon development along the arterial roads leading to the north and east and new housing estates. From 1966 sample census figures for the area we estimated that the total resident population was 127,000 and its age and sex and social class structure not statistically significantly different from that of England and Wales as a whole. Tables 1 and 2 show that there was a small excess of people over 44 years of age in the study area compared with England and

**TABLE 1**
*Age/Sex Distribution*
Percent distribution by age and sex of population in study area and in England and Wales estimated from 1966 Sample Census Data.

| AGE | MALES | | FEMALES | | TOTAL | |
|---|---|---|---|---|---|---|
| | Study Area | England + Wales | Study Area | England + Wales | Study Area | England + Wales |
| | % | % | % | % | % | % |
| 0—4 | 7 | 9 | 6 | 8 | 7 | 9 |
| 5—14 | 13 | 15 | 11 | 14 | 12 | 15 |
| 15—44 | 41 | 41 | 37 | 38 | 39 | 39 |
| 45—64 | 28 | 25 | 28 | 25 | 28 | 25 |
| 65+ | 11 | 10 | 19 | 15 | 15 | 12 |
| TOTAL 100% | 60.5 x $10^2$ | 22.8 x $10^5$ | 66.2 x $10^2$ | 24.3 x $10^5$ | 126.8 x $10^2$ | 47.1 x $10^5$ |

$x^2$ for males to females not significant at 5% level.
$x^2$ for total age distribution not significant at 5% level.

**TABLE 2**
*Social Class*
Percent distribution by social class[1] of population in study area and in England and Wales estimated from 1966 sample census data.

| SOCIAL CLASS | STUDY AREA | ENGLAND AND WALES |
|---|---|---|
| | % | % |
| I | 5 | 4 |
| II | 15 | 15 |
| III | 51 | 49 |
| IV | 17 | 20 |
| V | 9 | 9 |
| Not Classified | 3 | 3 |
| TOTAL=100% | 4678 | 1,623,208 |

[1]derived from 10% sample; males 15+ economically active or retired.
$x^2$ not significant at 5% level.

Wales and a small deficiency of social classes IV and V: neither of these differences was statistically significant at the 5% level.

**ACTIVE SEARCH FOR ACCIDENT VICTIMS**

We decided to attempt to monitor all home accidents in private dwellings which resulted in a person using medical care services. At a later date we aim to conduct a control study of home accidents where people do not use medical care services to supplement our initial findings. The basis of the primary data system is a continual active search. Staff employed by the Division continually contact the relevant medical care services to identify home accident victims. The system was established as follows.

**Hospitals**

The following hospitals have casualty departments that could receive patients from the study area:—

Southmead Hospital
Cossham Hospital
Bristol Royal Hospital
Bristol Royal Hospital for Sick Children
Bristol Dental Hospital
Bristol Eye Hospital
Frenchay Hospital

After a period of observation we found that virtually no persons attended the eye and dental hospitals after a domestic accident in the first instance. Patients treated in these hospitals first went to either of the two Royal Hospitals and were subsequently transferred. Therefore it was decided not to monitor these two specialist hospitals but to pick up the primary information about accidents involving teeth and eyes from other casualty departments.

After negotiations with the South West Regional Hospital Board, the United Bristol Hospitals and the relevant Medical Committees of the other hospitals, permission was obtained to monitor the casualty attendance registers and to obtain initial information regarding visits by domestic accident victims. An information card containing a brief description of the nature and purpose of the study and requesting public co-operation was produced and distributed in quantity to casualty reception desks to be given to injured persons or their relatives.

**General Practitioners**

Initial negotiations were commenced with the Executive Council who agreed to contact for us all the general practitioners who might be serving the population of the study area. Names of doctors were obtained and surgery addresses plotted on a map. There were 94 surgery addresses and 140 doctors. Each doctor was sent a copy of the research proposal and a letter asking for his co-operation. A nurse calls regularly at surgeries to collect names and addresses of victims of home accidents.

The practices were monitored for a period and as a result 41 doctors' names were deleted for the following reasons:—

| | |
|---|---|
| Very few patients from the study area | 13 |
| Complete refusal to co-operate or discuss proposals | 5 |
| Practice being run down, retirement or ill health | 2 |
| Surgery beside entrance to a major accident unit | 1 |
| Doctors interested but surgeries not attended by accident victims | 20 |

These 41 doctors worked from 31 surgeries which were removed from the nurses' visiting lists. In addition 6 surgeries used only a special clinic and 4 small branch surgeries which only opened for a few hours every week were also deleted, hence there were 99 doctors working from 53 surgeries who have been participating in the study and providing primary information for it.

**Fire and Ambulance Services**

Contact was made with the Chief Officers of these two emergency services and information regarding every call to an accident in the home was arranged to be passed on daily to the Research Division's Fieldwork Co-ordinator. Many of the notifications from both these sources were duplicated from casualty departments and general practitioners' surgeries. A weekly check of all notifications was carried out to identify such duplication.

**The Coroner**

One nurse fieldworker was allotted the special task of liaising with the Bristol Coroner's Officer who tells her of all deaths from home accidents within the City of Bristol. Frenchay Hospital is just outside the City limits in South Gloucestershire. Many victims of home accidents within the City and study area, die at Frenchay and those deaths are investigated by the South Gloucestershire Coroner's Officer whose office is just outside the City limits. He too provides information to us on deaths from home accidents of Bristol City residents. The study of fatal domestic accidents presents many social and procedural problems. There are also difficulties arising out of definition. For these reasons, and also because of their relative infrequency when compared with non-fatal accidents, a special study of all fatalities within the study is being carried out in parallel to the study of non-fatal accidents. The coroner has agreed to let us use all documents connected with the inquests but we make no special investigations until all formalities required by law have been completed.

The information network described above provides a continual flow of names and addresses of home accident victims to the Medical Research Division. The network is maintained by nurses employed for the study who continually contact and re-contact the medical care services involved, searching out the accident victims' records. The system does not merely rely on the medical care services notifying us. It is thus a positive search system rather than a notification system. The system is run by a Fieldwork Co-ordinator and an Administrative Assistant.

### PROCEDURE WITHIN THE HOME

Once access is gained to the home it is necessary to ensure that the visit is not unduly prolonged. Initially the victim is asked to give an account of what happened. When it is not possible to speak with the individual involved, details are obtained from another in the household who was present at the scene or who arrived immediately after the accident. This is often the spouse, or a parent or relative. The incident is usually well known within the household. On occasions when the person we met was not sure of what happened another visit was arranged to meet the victim personally. The recollection of accident details is often coupled with an invitation to see the exact site. If this is not forthcoming it is easy at this stage to ask for permission which is usually given. The description is then reinforced by reference to definite objects at the scene. At this point measurements and photography are begun.

All on site investigation is made by staff employed by the Health Education Council. The ideal number in the visiting team is three, one of whom has to be a doctor or nurse. It was decided early in the study that there should always be a female in the team as often there was only a woman and her children at home. If the team had more than three persons, investigations were hindered by over-crowding in the confines of the home. While the team's spokesman asks the questions, the other two help each other with measuring and photography and this minimises delay in completing the investigations.

### METHODS OF RECORDING DATA

Data concerning the accidents is recorded in writing and by photography.

(a) **Written Records**

In the earlier stage of the research the written records consisted mainly of a paraphrase of the main incidents in the accident sequence and its medical, social and economic consequence as related by the interviewee. In addition the date and time of the accident, its exact location and the name and age of any injured persons were noted. Then further questions were asked about the events preceding the incident and those which followed it. These included first aid measures, mode of transport to hospital and details of injuries and treatment. The questions asked depended on the nature of the incident. For example, where children were involved with harmful substances full details about the materials, its container, usual place of storage and harmful effects were included. After the first month, when about 100 accidents had been visited, and the types that were more frequent were apparent, it became possible to design special forms, for those particular types, covering aspects which appeared to be most often present. Accidents on staircases, suspected childhood poisoning and chip pan fires were three examples. The advice of a manufacturer of staircases was obtained in connection with that particular form. About the same time sufficient experience was gained of variables common to all types of accidents to enable a general form to be produced.

(b) **Photography**

Photographs were primarily intended to supplement the written descriptions but in fact they provided the means of recording places and objects in a form in which they could be repeatedly examined without returing to the scene and which captured technical details not at first recognised. This is most useful where specialist advice has to be obtained later.

There were several considerations which determined the type of photographic and other equipment chosen:

(a) Portability
(b) Simplicity; so team members without expert knowledge could produce good photographs
(c) Not requiring elaborate assembly and dismantling which would tend to minimise use, especially when time was short
(d) Needing minimum maintenance
(e) Operation in confined spaces (wide angle capabilities)
(f) Permit recording of close detail.

The photographic and other equipment chosen to be taken to accident sites is listed in the Appendix.

### ILLUSTRATIVE CASE STUDIES

The following case histories illustrate the importance of detailed on-site investigations and how the most unexpected results emerged from situations which would have been grossly mis-interpreted if we had relied on a report taken by a doctor in a surgery or at a casualty centre.

**Accident Number 045/033.** *Burns to face from gas cooker explosion.*

A notification was received that a school girl had been treated at a hospital for burns following an explosion in the gas cooker at home on 6.9.71. The home was visited and permission was obtained from the girl's father to investigate the accident. On the day of the accident his wife had cooked the lunch using the oven of the gas cooker which she had had for about three years. When her daughter aged fifteen years came home from school during the lunch break she served the meal and turned off the oven. After the meal, which was eaten in a room adjacent to the kitchen and took about half an hour, she lit one gas ring and put a kettle of water on to make some tea. She then asked her daughter to replace in the oven the food that was remaining from the meal. When the girl opened the oven door there was an explosion which hurled her across the kitchen. She suffered from first and second degree burns to the face with singeing of her hair, eye-lashes, and eye-brows. When the mother ran in from the adjacent room she saw her daughter on the ground with her hair on fire. The husband was home at the time and together they managed to extinguish the flames, and telephone for an ambulance which took the girl to hospital. She received treatment in the casualty department but was not admitted.

The cooker a Flavel Mark II had been bought on hire purchase from the South Western Gas Board in November 1968 and at the time of the accident the Gas Board was still responsible for its maintenance. The controls of the cooker were in front

Plate X. Flavel Mark II Gas Cooker involved in accident 045/033.

**Plate XI. Plastic oven control knob of gas cooker showing the sleeve and encircling steel spring. Note the missing portion of plastic resulting from the 'edge failure' described in the text.**

of the hob at waist height (Plate X). Each control consisted of a plastic disc with a sleeve which fitted onto a metal spindle. The sleeve and the spindle were surrounded by a steel spring which held the two components together. This spring is seen in Plate XI. During the three years that the cooker had been in use, a total of eight control knobs had to be replaced due to breakage. Two broke in the first year of use, and three in each of the second and third years. In each instance the breakage occurred to the plastic of the sleeve. As a result of these breakages the fit between the sleeve and the spindle became loose and consequently the housewife lost control of the gas flow to that particular burner. Every time a knob broke the Gas Board was informed. A fitter readily attended and replaced the knob. She was charged a few shillings for each replacement. When disgust was expressed at these repeated breakages the owner was told that his wife used too much force when turning the knobs. The official of the Gas Board mentioned that there were hundreds of cookers with similar knobs giving no trouble at all A few weeks before the accident in question, the control knob of the oven had similarly failed. Plate XI shows the broken sleeve of the oven control knob. It is evident that the plastic has sheared at the edge where it was in contact with the metal spindle. This is called 'edge failure'. Plates XII and XIII show the performance of this broken knob on its spindle. The two photographs were taken at the extreme positions to which the knob could be turned, in the anti-clockwise and clockwise directions respectively, without effecting any movement in the spindle.

An immediate replacement could not be obtained but meals still had to be cooked until the Gas Board attended. During this period the housewife had to gauge by feel the position of the knob controlling gas to her oven. In her own words she had to "keep twiddling the knob back and forth" until she felt the spindle engage and turn. This was the procedure which she had adopted when she turned the oven off after having cooked the meal on the day of the accident. With such a procedure it was quite possible for her to have first extinguished the oven and then to have inadvertently turned the gas on once again. As a result gas could have slowly accumulated within the oven during the time the family were having their meal and when the girl went to replace food in the oven it is possible that explosive combustion of the

**Plate XII. Oven control knob in the extreme anti-clockwise position. Compare this with plate XIII showing extreme clockwise position of free movement due to loose fit on the spindle.**

**Plate XIII. Oven control knob in extreme clockwise position of free play.**

gas occurred from the lighted burner under the kettle.

It was evident from the details of the events that led up to the explosion that no members of the family who participated in those events could be blamed for carelessness. The escape of gas and the consequent explosion could be very clearly attributed to failure of the plastic material used in the manufacture of the control knob. Several possibilities had now to be considered. Examination of the broken plastic showed that it had sheared and crumbled where it had been in contact with the metal spindle. Excessive forces, more than the plastic could bear, might in fact have been due to a defect within the mechanism of the tap making the spindle more difficult to turn. The extra force that would then have been needed to operate the control could have produced excessive shearing forces between the spindle and the plastic sleeve. On the other hand if the mechanism of the tap were not defective, then the only other reason for the failure had to be a weakness of the plastic itself.

**Plate XIV. Control knobs from other gas appliances showing construction of sleeves from nylon (right) and metal (left).**

Plate XIV shows the control knobs from two other gas appliances. Both have sleeves designed to fit into spindles of a similar shape to those in the Flavel gas cooker. However in one the sleeve is made of metal and in the other it is made of nylon, while in both, those parts which are handled are made of white plastic material. In both these instances it seems that the manufacturers have recognised the special need for these sleeves to be of a stronger material. The Department of Chemistry of the University of Bristol was contacted in an endeavour to learn something about the types of plastic materials and their uses. The Department put the Medical Research Division in touch with the plastics division of a firm of international repute. Following a telephone conversation the damaged control knob was sent to them. A few days later they informed us that the material used in its construction was a thermo-setting resin based on urea formaldehyde. The material was widely used for electrical household fittings and all types of switches. It had a high impact strength but was well known for brittleness. It could very defintely not perform the task in this particular control knob. The plastic would crumble. We were told that the manufacturers had recognised this problem and some were now exclusively using glass reinforced nylon for this purpose. The 'edge failure' which had occurred on this particular control knob would thus be eliminated. In the opinion of the member of the plastics division who spoke to us this particular manufacturer had apparently given little thought to the properties of this material in relation to the task it had to perform.

**Accident Number 028/054.** *Fractured foot in fall from chair.*

The Medical Research Division was informed that a housewife aged 39 was treated in hospital for a fractured bone in the foot following a fall from a chair. We visited the home and interviewed the woman.

**Plate XV. Ceiling lamp in living room showing bayonet lamp socket adaptor in accident 028/054.**

The fall occurred in the living room when the housewife stood on a chair to attach her vacuum cleaner to a two-way bayonet adaptor which was permanently connected to the flex of the ceiling lamp (Plate XV). She always plugged her cleaner into the ceiling lamps not only in the living room but also in the bedrooms. In the bedrooms she did not have adaptors but she removed the bulb and connected the cleaner to the lamp socket, replacing the bulb when she finished. The family had rented the house from the Bristol City Corporation for five years and during that entire period she had used her vacuum cleaner in this way. We asked why she used the cleaner with bayonet adaptors. We learned that *all the power points in the house were of the round pin variety and differed in size throughout the rooms.* The distribution of sockets was as follows:—

Living room—one 5 amp socket
Dining room—one 15 amp socket
Kitchen—two 5 amp sockets
3 Bedrooms—one 15 amp socket in each room.

**Plate XVI. Three round pin power sockets of different amperage showing variation in size.**

As the size of round pin sockets varied with amperage (Plate XVI) it would have been impossible for her to use her vacuum cleaner around the house unless she changed the plug for each room. She therefore fitted a two pin adaptor to the cleaner and used the lamp sockets. During the five years this family were in the house they had not asked the Corporation to change the power sockets, though the housewife told us she thought "the Council should change the plugs in the house".

About a fortnight later a reported accident involving a fall (accident number 031/010) led us to another house in the same neighbourhood where the sockets had recently been changed by the Council. The details of this accident were as follows:—

A girl of 14 had fallen from a garden shed. While the family was being interviewed the nurse fieldworker noticed a tangle of electric wires in a corner of the living room (Plate XVII). Upon further enquiry it was learned that the wires were part of a junction box system distributing power from the single 5 amp round pin socket in the living room to the radio, television and other electrical appliances used there. As the tangle of wires appeared to constitute a hazard the fieldworker mentioned it in her report to the Division. The house was visited and the following distribution of power outlets by amperage were found.

Living room—one 5 amp socket
Kitchen—two 5 amp sockets
one 15 amp socket
Dining Room—one 15 amp socket
3 Bedrooms—one 15 amp socket in each room

*All these sockets were of the round pin variety* and size varied with the amperage.

The family had moved into the house six months previously, in November 1970. It had been vacant for

**Plate XVII. Tangle of electric flexes on living room floor arising from junction box arrangements. Accident 031/010.**

two weeks prior to that, after the previous tenants had left. It was owned by the Corporation. The new tenant had noticed that the power sockets were cracked, loose and in a generally dangerous condition. He therefore requested that new sockets be fitted during the time the house was vacant before his family moved in. These were eventually fitted but they were of the round pin variety and therefore varied in size. This left a problem facing the housewife with regard to the use of her portable appliances and resulted in the use of junction boxes involving tangles of wires. We have since learnt that notwithstanding the existence of BS. 1363 relating to the single sized, square pin, 13 amp plug and socket, round pin sockets are still being installed by the Electricity Board where round pin equipment is being replaced in houses belonging to the Corporation.

*Two Instances Involving Children with Harmful Substances*

**Accident Number 003/034**

Notification was received that a 3 year old had been admitted to hospital following the ingestion of a quantity of shoe dye. His stomach was washed out in casualty.

When the house was visited the mother said that child had been found by her with the open bottle of black liquid polish. The child was daubed all over including its face but she saw no staining inside the mouth. The child was not suffering any ill effects but she took the child to the hospital. At the hospital she was told that the child had not actually swallowed any. The hospital notes recorded that the child was fully conscious and alert. Nothing abnormal was found on examination. The stomach was washed out and the contents were not coloured. He was admitted for one night. The incident was eventually classified as a case of 'poisoning'.

**Accident Number 003/030**

A child aged 3 years was taken to hospital after taking 2 Panadol tablets three quarters of an hour earlier. The mother had made her vomit. She had no abnormal physical signs on examination. The stomach was washed out and the return water was clear. The child was admitted for one night. The case was classified by the hospital as a case of 'poisoning'. When the home was visited the mother said she had found the child with the open screw cap bottle containing the tablets. She said the child took two tablets and spat out some fragments.

These two are typical of the majority of cases of children being involved with substances not intended for them. The visits to the home and interviews with parents indicate that where the children have no harmful effects when first examined there seems to be a strong evidence from the scene that nothing very much was ingested. A large proportion of the total cases belong to this category; many of these are in fact admitted and help to swell the national statistics of cases of 'poisoning'. A detailed report is being prepared covering all aspects of childhood poisoning.

### TWO STAGE VISITING

After studying some 200 accidents in depth over a period of 18 weeks it became clear that although some patterns were emerging it was not possible to continue the study on the basis of studying all notifications in that way. There were other research topics for the Medical Research Division to develop; we had not enough specialists to examine all technical problems of obvious relevance in the cases and we were begining to lose sight of the wood for the trees. We therefore re-organized the study into a two stage system of visiting. We wanted to preserve the home visit as the primary means of identifying the phenomena involved

**TABLE 3**

*Age and Sex Incidence*

Estimated incidence of persons seeking medical care after a home accident, per thousand population per year in the study area,[1] by age and sex; two studies 1970/71.

| AGE | STUDY 1 Nov. 1970—March 1971 | | | STUDY 2 May—Nov. 1971 | | | Estimate of Incidence for year 1970/71 from two studies combined | | |
|---|---|---|---|---|---|---|---|---|---|
| | Males | Females | Total | Males | Females | Total | Males | Females | Total |
| 0 — 4 | 71 | 83 | 76 | 62 | 46 | 54 | 63 | 51 | 58 |
| 5 —14 | 12 | 13 | 13 | 22 | 15 | 19 | 21 | 14 | 18 |
| 15 —44 | 11 | 13[2] | 12 | 8 | 12 | 10 | 8 | 13 | 10 |
| 45 —64 | 9 | 19 | 14 | 5 | 14 | 9 | 5 | 15 | 10 |
| 65+ | 12 | 25 | 21 | 13 | 20 | 17 | 13 | 20 | 18 |
| ALL AGE GROUPS | 15 | 21[2] | 18 | 14[3] | 17[4] | 15 | 14 | 17 | 16 |
| Number of Cases | 77 | 118 | 195 | 397 | 529 | 926 | 474 | 647 | 1121 |

[1] Estimates of population of study area derived from age sex distribution in study area for 1966 Sample Census, adjusted for total population change in Bristol CB 1966 Sample Census—1971 Census.
[2] Includes one woman estimated to be 30-34 years who refused to give her age.
[3] Includes 3 men who refused to give their age.
[4] Includes 7 women who refused to give their age.

in the accident — we had fundamental reservations about the alternative approach of the US surveillance system on product safety where a clerk attempts to observe the domestic products and environment involved in the accident from his seat in the emergency department of the hospital (National Commission Product Safety 1970). We therefore recruited and trained over an extended period a group of qualified nurses employed by us to make initial visits to all the home accident sites in the study area from which notification had been received through the same information network previously described. A standard form was developed for recording basic information which would provide a general picture of the frequency, severity and cost of accidents and the pattern of them over time.

The results collected in this way were then used as a sampling frame for specialist follow up visits. Thus the specialist staff were reserved for particular types of accidents and in this manner we have been conducting special investigations of carbon monoxide poisoning from gas water heaters, fires involving fat pans, poisoning of children, falls from ladders and steps, scalds from kettles, and other special types of accident.

Fieldwork continues; we have visited over 3,000 home accidents, 500 of these being investigated in depth. We are preparing a series of reports on the general findings and on the special studies. A brief summary of the numerical findings on the first two study periods is shown in tables 3-6.

### RESULTS

One of the primary objectives of our studies has been to establish measures of the incidence and social and economic impact of home accidents. From results of the first thousand cases we estimate that there are 16.3 home accidents per 1000 population per year; of which 15.5 involved medical care services and 0.8 visits by the Fire Brigade alone. We also estimate from the Registrar General's figures that there are 0.1 deaths per 1000 population per annum as a result of home accidents.

From our measurements of the social and economic impact of home accidents we estimate that for a population of 1000 persons in a year home accidents result in 24 days of in-patient care, 35 attendances in hospitals as out-patients, 6 ambulance journeys, 7 general practitioner consultations, 130 days of restriction of economic, domestic, educational and social activity. From the age at death and using the Registrar General's Life Tables we estimate that there are three life years lost per 1000 population per year through home accidents. If we multiply up these figures for a population of 50 million persons (or our model country) then we find that in a year there are 820,000 home accidents resulting in 1.2 million in-patient days, 1.8 million hospital attendances, 300,000 ambulance journeys, 400,000 general practitioners' consultations, 6.5 million days of restricted activity and 132,000 life years lost through premature death (See table 4).

For a population the size of Bristol (426,000) we estimate that in a year there are 6,900 home accidents resulting in 10,200 days of in-patient care, 14,900 attendances at hospital out-patients, 2,600 ambulance journeys, 3,000 general practitioner consultations and 58,400 days of restriction of economic, domestic, educational and social activity. Using a method of classifying injury severity previously developed (Kolbuszewski 1969) we found that 3% of the persons seeking medical care after a home accident were found to have no injury on examination by a doctor; 63% were found to have minor injuries; 28% were found to have moderate injuries and 6% were found to have severe injuries.

There are a variety of methods of measuring the costs of home accidents. It is necessary to measure the cost of components of home accidents in order to arrive at a sum which might be an acceptable amount for society to spend on avoiding the social and economic suffering caused by home accidents. In estimating the cost it must be realised that, with the exception of bus fares and prescription charges, none of the measures are manifested as physical cash flows. The method of assessing cost is the subject of a separate paper (Roberts 1972) but a summary of the figures is given in tables 5 and 6).

We estimate that the total cost of home accidents is £2,600 for 1,000 population per year; of which £2,000 is attributed to the cost of premature death, £300 to the cost of medical care services, £100 to the cost of property damage, £100 to the cost of restriction of economic, domestic, educational and social activity and less than £100 to other costs, including the use of other public services and the use of private transport in obtaining medical care. For a population of 50 million we estimate that an annual £15 million cost for medical care falls directly upon the Department of Health and Social Security. This figure consists of £9.5 million for hospital in-patient care; £4.3 million cost of hospital out-patient care; £0.4 million cost for ambulance services; £0.6 million cost to the general practitioner services; other items of medical care not listed amount to £0.1 million. In addition to the annual medical care costs we estimate that for a population of 50 million persons the cost of property damage is £3.9 million. The cost of restricted activity including economic, domestic, educational and social activity is £6.9 million, and there are £14 million other costs. We have attributed a further £100 million to the cost of premature death from home accidents—that is about £10,000-£15,000 per death.

For a population the size of Bristol we estimate that the costs of home accidents in a year are £1,100,000 total cost of which £852,000 represents a cost for loss of life, £126,000 the cost of medical care services, £33,000 the cost of property damage, £58,000 the cost of restrictions of economic, domestic, educational and social activity and £8,000 other costs.

The above costs may be seen as measures of the potential benefit to the community from eradicating home accidents. They confirm that home accident prevention is a major problem. By examining the distribution of costs associated with potential accident types they have immediate use in identifying priorities for prevention and treatment programmes, for focusing on topics for further research and development and for putting the problem of home accidents in a perspective with other health problems.

Further reports are in preparation which will give details from our case study material and special studies.

**TABLE 4**

*Estimated Morbidity from Home Accidents*

Estimated incidence of use of medical care services, of restricted activity days, and of life years lost from premature death, by severity of injury found on examination of persons seeking medical care after a home accident; and estimated annual total frequency of items for 50 million population; two studies Bristol 1970-71.

| Items | Incidence per thousand population year | | | | | | | Estimated annual frequency of items for population of 50 million persons |
|---|---|---|---|---|---|---|---|---|
| | Persons killed and persons seeking medical care by severity of injury. | | | | | | | |
| | No Injury | Minor Injury | Moderate Injury | Severe Injury | Fatal Injury | Injury Severity not known or not recorded | All persons killed or seeking medical care | (millions) |
| No. patient days | — | 12 | 3 | 8 | — | — | 24 | 1.2 |
| No. attendances at hospital as out-patient | — | 14 | 16 | 3 | — | — | 35 | 1.8 |
| No. ambulance journeys | — | 1 | 3 | 1 | — | — | 6 | 0.3 |
| No. of G.P. visits | — | 4 | 2 | 1 | — | — | 7 | 0.4 |
| No. days of restricted activity | — | 43 | 51 | 35 | — | — | 130 | 6.5 |
| Life years lost | — | — | — | — | 3 | — | 3 | 0.1 |

**TABLE 5**

*Total Costs of Accidents in Study Area*

Total percent and mean costs of accidents involving persons seeking medical care, by type of cost Study 1 November 1970—March 1971.[1]

| Cost Type | Total Cost £ | Total Cost as % of Aggregate Cost % | Mean Cost Per Person Seeking Medical Care £ |
|---|---|---|---|
| **Medical Care Costs** | | | |
| Inpatient costs | 2364 | 41 | 12.1 |
| Outpatient costs | 1080 | 19 | 5.5 |
| Ambulance costs | 98 | 2 | 0.5 |
| GP costs | 138 | 2 | 0.7 |
| Other Medical Care Costs | 30 | 1 | 0.2 |
| Sum of Medical Care Costs | 3709 | 64 | 19.0 |
| Property damage costs | 255 | 4 | 1.3 |
| Restriction of production, education and social activity costs | 1724 | 30 | 8.8 |
| Other costs | 136 | 2 | 0.7 |
| Aggregate Costs | 5826 | 100 | 29.9 |

[1] Deaths and the cost to the community of deaths from home accidents are excluded from this table.

### TABLE 6

*Estimated Cost of Home Accidents*

Estimated cost incidence of home accidents including home accidents involving medical care services and home accidents involving fire service but not medical care services, by severity of injury found on examination and by component costs; and estimated annual total costs of home accidents for 50m. population; two studies 1970-71.

| Component costs of home accidents | Costs per thousand population per year—(Costs in £ for all component costs) — Persons killed and persons seeking medical care by severity of injury: No Injury | Minor Injury | Mod. Injury | Severe Injury | Fatal Injury | Injury Severity N.K. or N.R. | All persons killed or seeking medical care | Accidents involving fire service but not involving medical care | All persons killed or seeking medical care and all accidents involving fire service but not involving medical care | Estimated total cost of home accidents for population of 50m/yr. £m. |
|---|---|---|---|---|---|---|---|---|---|---|
| 1. Inpatient Cost | — | 94 | 23 | 67 | — | — | 190 | 0 | 190 | 9.5 |
| 2. Outpatient Cost | — | 35 | 39 | 7 | — | — | 86 | 0 | 86 | 4.3 |
| 3. Ambulance Cost | — | 2 | 4 | 1 | — | — | 8 | 0 | 8 | 0.4 |
| 4. G.P. Care Cost | — | 6 | 3 | 1 | — | — | 11 | 0 | 11 | 0.6 |
| 5. Other Medical Care Cost | — | 1 | 1 | 1 | — | — | 3 | 0 | 3 | 0.1 |
| Total Costs 1-5 | 1 | 138 | 70 | 77 | — | — | 298 | 0 | 298 | 14.9 |
| 6. Property Damage Cost | — | 17 | 2 | 1 | — | — | 20 | 57 | 77 | 3.9 |
| 7. Restricted Activity Cost | — | 46 | 54 | 37 | — | — | 138 | 0 | 138 | 6.9 |
| 8. Other Costs | — | 5 | 4 | 2 | — | — | 11 | 8 | 19 | 1.0 |
| Total Costs 1-8 | 1 | 200 | 128 | 116 | — | — | 467 | 65 | 532 | 26.6 |
| 9. Premature Death Cost | 0 | 0 | 0 | 0 | 2,018 | — | 2,018 | 0 | 2,018 | 100.9 |
| Total Costs 1-9 | 1 | 200 | 128 | 116 | 2,018 | — | 2,485 | 65 | 2,550 | 127.5 |
| | | | | | | | | | | Total Annual Home Accidents |
| Incidence of home accidents per 1,000 pop. per year | 0.4 | 9.8 | 3.6 | 0.9 | 0.1 | 0.9 | 15.6 | 0.8 | 16.4* | 820,000 |

*There are 16.3 accidents per 1,000 population per year; but when accidents involving more than one person are counted by the numbers of persons seeking medical care the figure is as stated.

**ACKNOWLEDGEMENTS**

Any opinions expressed in this paper are those of the authors and not necessarily those of the Council. The authors acknowledge the work of Dr. J. W. Dale and staff of the Medical Research Division of the Health Education Council in the current Bristol study of home accidents, the co-operation of the local hospital and health authorities, general practitioners, the ambulance and fire services, the coroner and the victims of the home accidents without whom this paper could not have been written.

The authors also wish to acknowledge the help and encouragement that has been given to the Medical Research Division in the investigation of home accidents by the Department of Health and Social Security, the Home Office, the Medical Commission on Accident Prevention and the Royal Society for the Prevention of Accidents.

All enquiries on this paper should be addressed to:

Mr. J. L. Roberts, B.Soc.Sc., M.Sc., A.H.A.,
Medical Research Division,
Health Education Council,
Canynge Hall,
Whiteladies Road,
Bristol BS8 2PR.

who is at present co-ordinating the home accident studies.

### REFERENCES

Backett E. M. (1965). Domestic Accidents (World Health Organisation).

De Haven H. (1942). Mechanical Analysis of Survival in Falls from Heights of Fifty to One Hundred and Fifty Feet. War Medicine 2 586-596 American Medical Association.

De Haven J. (1952). Accident Survival—airplane and passenger automobile. Paper presented to Annual Meeting of the Society of Automobile Engineers. January. In Haddon W. et al. (1964) op. cit. p. 562-568.

Haddon W., Suchman E. A. and Klein D. (1964). Accident Research, Methods and Approaches. Harper and Row.

Hasbrook A. H. (1953). Crash Survival Study. National Airlines DC 6 Accident. Informative Accident Release 15th Oct. 1953. Crash Injury Research, Cornell University Medical College, New York.

Jamieson K. G., Duggan A. W., Tweddell J., Pope L. I., Zvirbulis V. E. (1971). Traffic Crashed in Brisbane, A.R.R.B. Special Report No. 2.

Kolbuszewski J., Mackay G. M., de Fonseka C. P., Blair I. and Clayton A. B. (1969). The Causes and Effects of Road Accidents Vol. I-V, Department of Transportation and Environmental Planning, Publication No. 33, University of Birmingham.

Medical Commission on Accident Prevention (1970). Seventh Annual Report 1970-1971 London p. 17.

National Commission on Product Safety (1970). Final Report and reports of hearings. U.S. Government Printing Office and Bureau of Product Safety, Bethesda, Maryland.

Roberts J. L. (1972). Frequency, severity and costs of Home Accidents. Unpublished research paper. Health Education Council.

Robertson J. S., McClean A. J., and Ryan G. A. (1966). Traffic Accidents in Adelaide, South Australia. Australian Road Research Board, Special Report No. 1.

States J. D. and States D. J. (1968). The Pathology and Pathogenesis of Injuries Caused by Lateral Impact Accidents. Proceeds of the Twelfth Stapp Car Crash Conference, p. 72. Society of Automotive Engineers, New York.

### APPENDIX

**List of Photographic and Other Equipment Used in Detailed 'On Site' Investigations of Home Accidents**

Exa 5000, single lens reflex 35 mm camera body

Exakta VX 1000, single lens reflex 35 mm camera body

Tessar f 2.8, 50 mm lens, Exakta fitting

Soligor f 2.8, 28 mm lens, Exakta fitting

Macablitz 182 rechargeable electronic flash

Close-up lenses 1D, 2D, and 3D on 49 mm screw mounts

Weston Master V exposure meter

125 ASA black and white film

64 ASA colour slide film

All this equipment was easily carried in a small bag. The only maintenance necessary was periodical recharging of the flash battery. A supply of spare film was always kept in the carrying case. An 18 inch rule with bold markings in black and white was available as a scale to be placed near articles being photographed. This rule has now been changed to metric values.

In addition to the above photographic equipment the following apparatus was also available for use at the scene:

| | |
|---|---|
| Metre Tape | Screw Driver |
| 2 Metre Tape | Draeger Normalair Gas Detection Pump |
| 500 mm Rule | Draeger Detection Tubes for CO and $CO_2$ |
| Outside Callipers | Hot Air Probe for Above Pump |
| Inside Callipers | Graduated Triangle |
| Weston Illumination Meter | Weighing platform |
| Inclinometer | |
| 3 kg spring balance | |
| Scissors | |

## Figures and Tables

**Plate V. f1:**
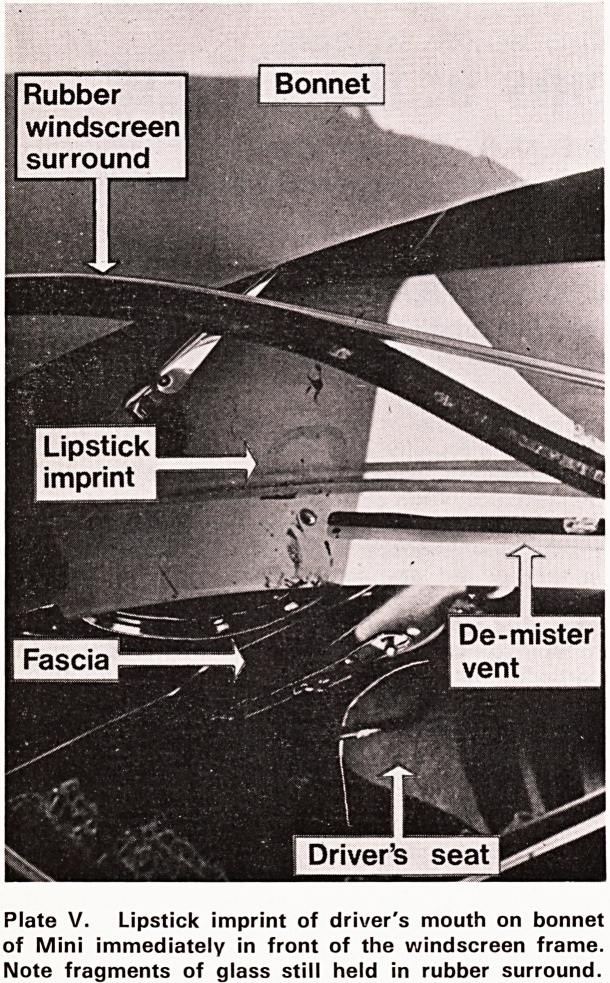


**Plate VI. f2:**
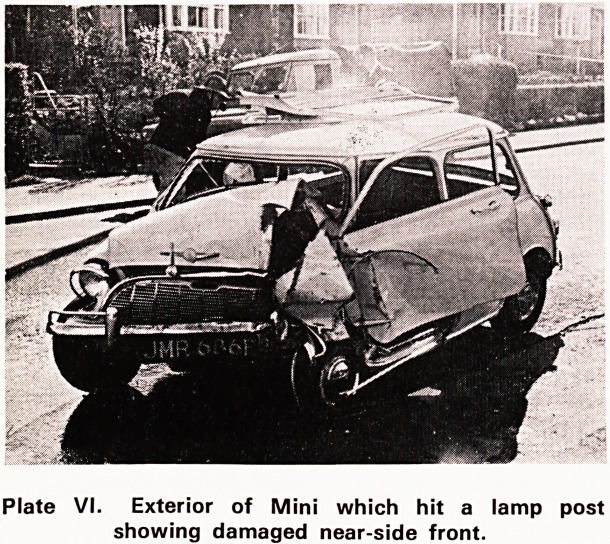


**Plate VII. f3:**
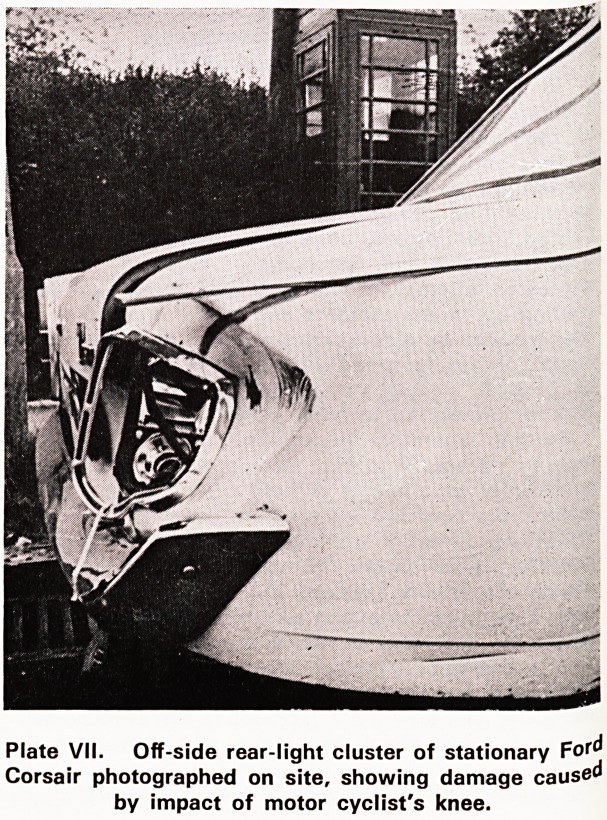


**Plate VIII. f4:**
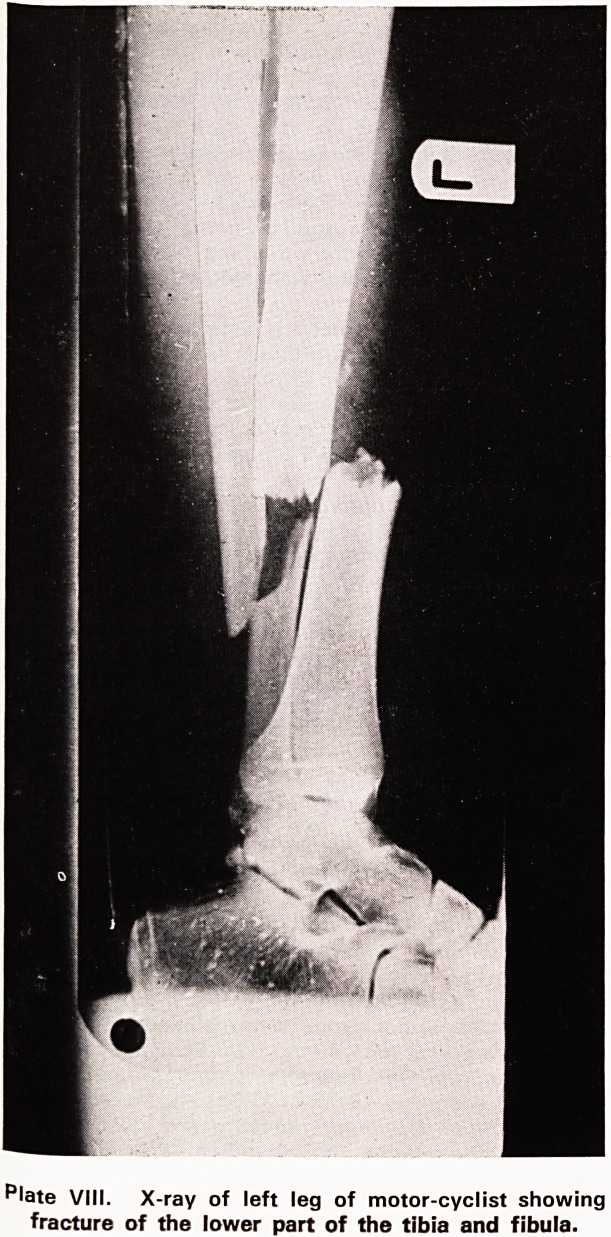


**Plate IX. f5:**
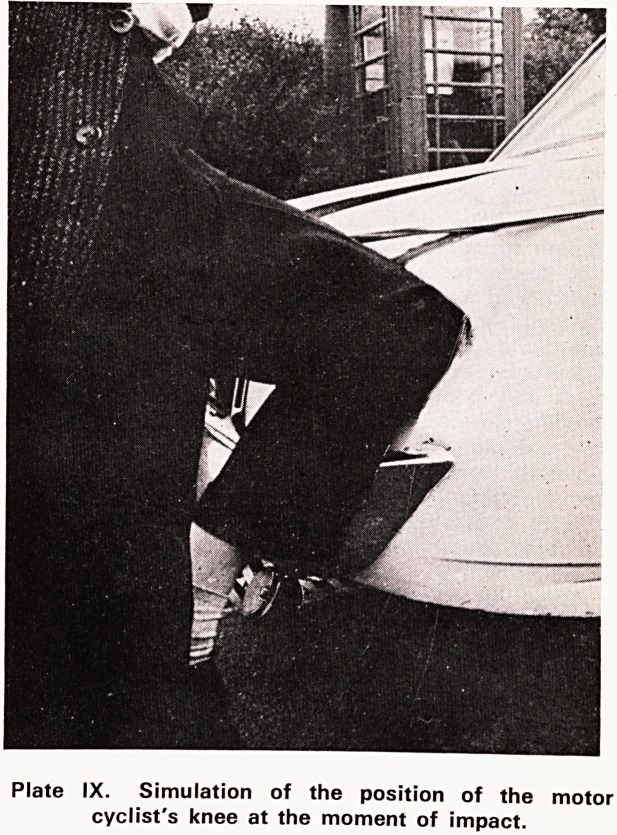


**Plate X. f6:**
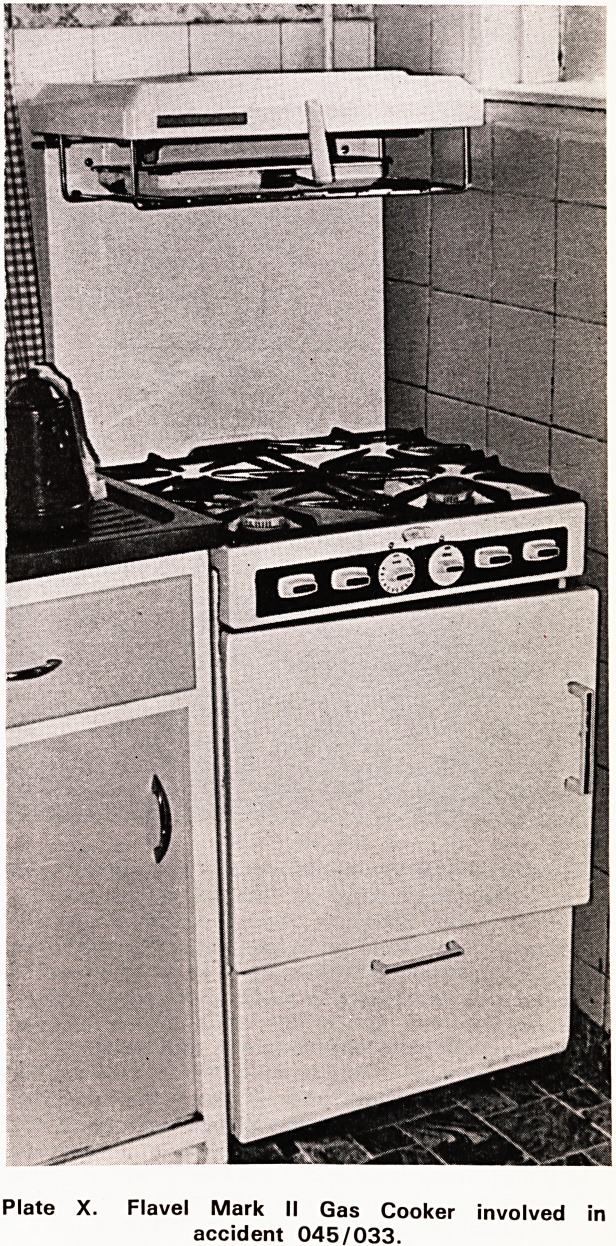


**Plate XI. f7:**
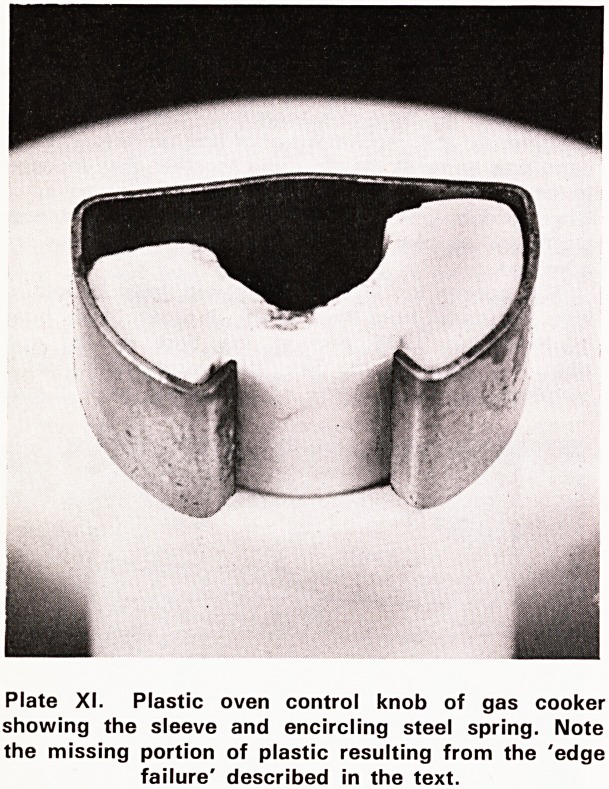


**Plate XII. f8:**
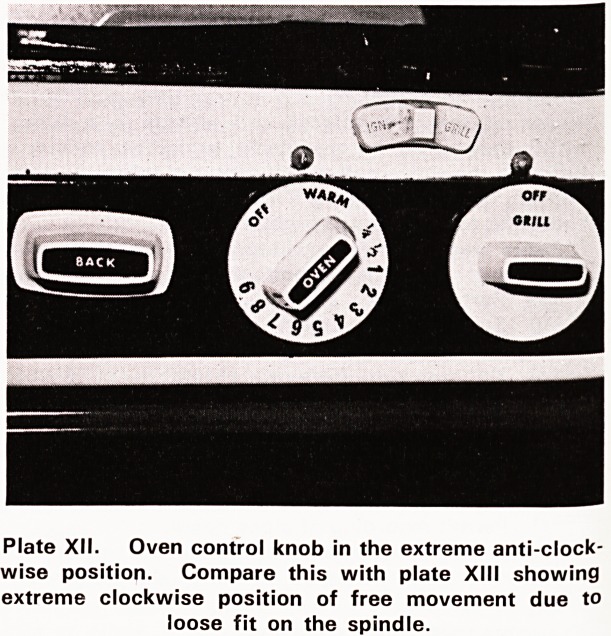


**Plate XIII. f9:**
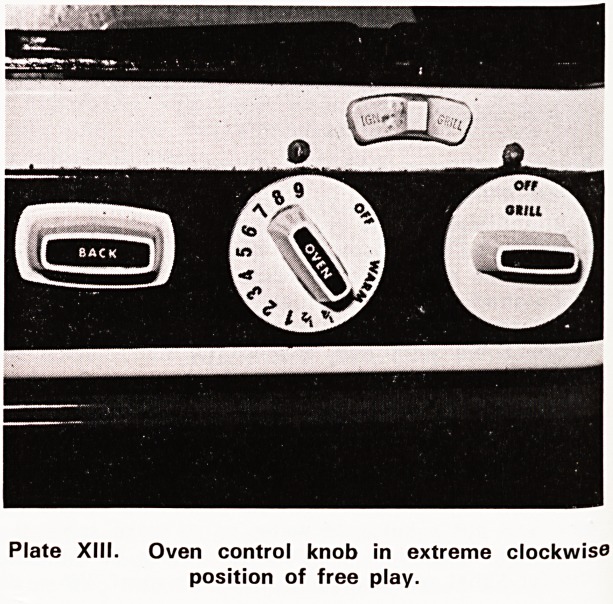


**Plate XIV. f10:**
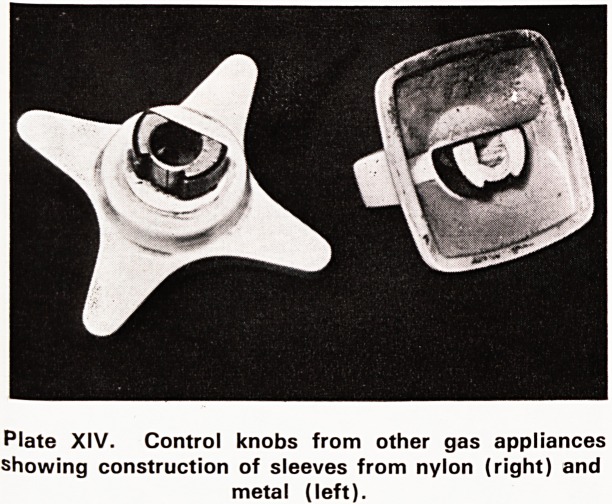


**Plate XV. f11:**
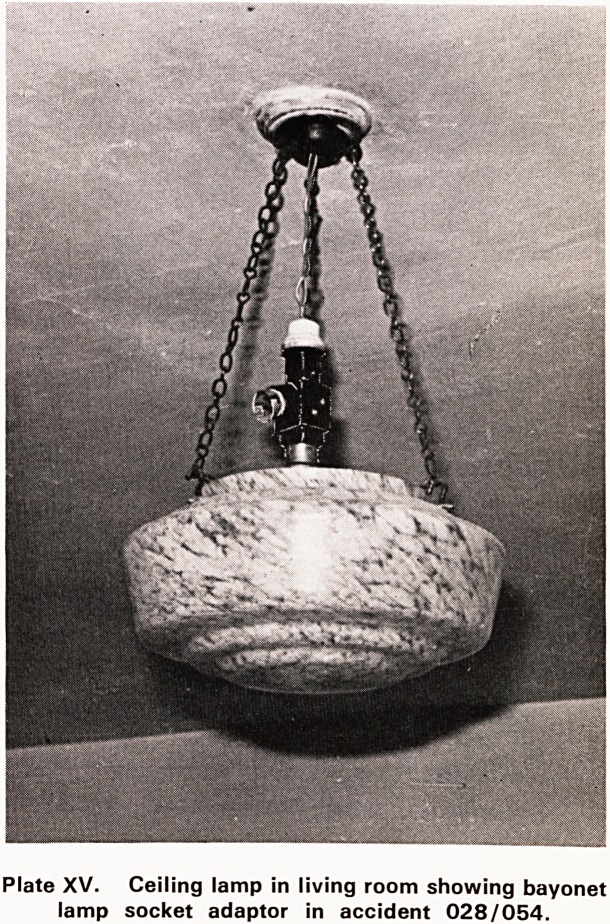


**Plate XVI. f12:**
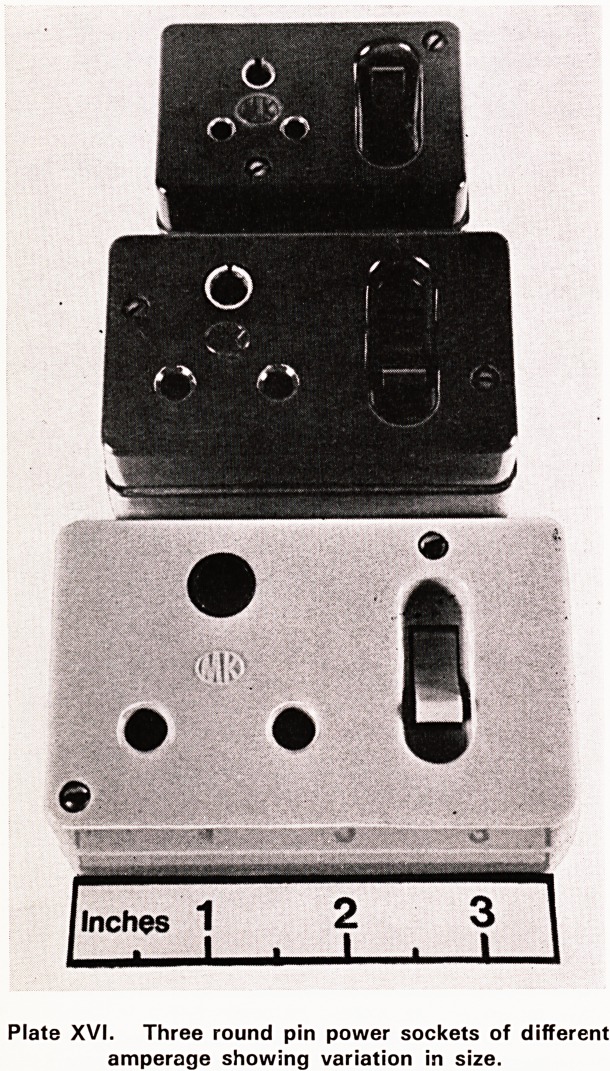


**Plate XVII. f13:**